# Risiko für Postinjektionssyndrome nach Vergabe von Olanzapin-Depot: Ergebnisse einer Auswertung von Real-World-Daten an einer psychiatrischen Universitätsklinik mit Vollversorgungsauftrag

**DOI:** 10.1007/s00115-024-01733-2

**Published:** 2024-09-17

**Authors:** Wolfgang Strube, Elias Wagner, Andreas Gartenmaier, Antje Grünemeyer, Klaus Peter Schmelzer, Alkomiet Hasan

**Affiliations:** 1 Klinik für Psychiatrie, Psychotherapie und Psychosomatik, Medizinische Fakultät, Universität Augsburg, 86156 Augsburg, Deutschland; 2 Evidenzbasierte Psychiatrie und Psychotherapie, Medizinische Fakultät, Universität Augsburg, Stenglinstraße 2, 86156 Augsburg, Deutschland; 3 Bezirkskliniken Schwaben, Apotheke des Bezirkskrankenhauses, Lindenallee 2, 89312 Günzburg, Deutschland; 4 Apotheke, Universitätsklinikum Augsburg, Stenglinstraße 2, 86156 Augsburg, Deutschland; 5 DZPG (Deutsches Zentrum für Psychische Gesundheit), Partnerstandort München/Augsburg, Augsburg, Deutschland

Die Wirkstoffformulierung Olanzapinpamoat ist in Deutschland seit November 2008 zugelassen für die Erhaltungstherapie von Erwachsenen mit einer Schizophrenie, die durch eine Akuttherapie mit oralem Olanzapin hinreichend stabilisiert werden konnten. Das sogenannte Postinjektionssyndrom (in der englischen Terminologie „postinjection delirium/sedation syndrome“, PDSS) stellt eine Komplikation nach Verabreichung von Olanzapin als Depotdarreichungsform Olanzapinpamoat dar. Klinisch ist das PDSS durch Symptome eines Delirs, insbesondere durch Orientierungsstörungen, Bewusstseinsstörungen, Verwirrtheit, Aufmerksamkeitsstörungen und intermittierende psychomotorische Unruhe sowie durch eine Sedierung unterschiedlichen Schweregrades – in seltenen Fällen bis hin zum Koma – charakterisiert [3]. Betroffene sind beim Auftreten eines PDSS somit besonderen medizinischen Folgerisiken durch die mögliche Schwere der Symptomatik ausgesetzt. Daher ist in der Fachinformation hervorgehoben, dass nach Vergabe von Olanzapinpamoat behandelte Personen über einen Überwachungszeitraum von 3 h hinweg auf Anzeichen eines PDSS nachbeobachtet werden sollen, um ein Auftreten möglichst frühzeitig zu erkennen [6]. Für Behandelnde ist es daher bei der Aufklärung über die Risiken einer geplanten Behandlung mit Olanzapin als Depot von besonderer Bedeutung, die Häufigkeiten und Risikofaktoren zu kennen, mit denen diese Nebenwirkung auftreten kann.

## Bisherige Datenlage aus klinischen und retrospektiven Studien

Zum einen liegen wissenschaftliche Auswertungen darüber vor, wie oft das PDSS als Nebenwirkung in klinischen Studien beobachtet wurde. Dabei wurden basierend auf mehr als 45.000 Olanzapindepotinjektionen, die mehr als 2000 Patienten verabreicht wurden, Inzidenzraten von 0,07 % über alle Injektionen hinweg gemittelt und ein kumulatives Risiko von 1,85 % über den Behandlungsverlauf der Patienten mit mehreren Injektionen hinweg ermittelt [5]. Zum anderen liegen Ergebnisse retrospektiver Übersichtsarbeiten vor, die über 24 europäische Länder hinweg und basierend auf über 103.000 Olanzapindepotinjektionen über einen Zeitraum von 5 Jahren geringere Inzidenzraten von 0,044 % über alle Injektionen gemittelt sowie ebenso niedrigere kumulative Risikoraten von 1,17 % über den Behandlungsverlauf von 3858 Patientinnen und Patienten berichteten [1, 2, 4]. Dabei wurden nur bei einem einzigen männlichen Patienten insgesamt 2 PDSS-Ereignisse innerhalb eines Behandlungsverlaufs berichtet. Zudem konnte gezeigt werden, dass sich die PDSS-Symptomatik in mehr als 90 % der Fälle innerhalb der ersten Stunde nach Gabe der Injektion entwickelte, womit die meisten PDSS-Ereignisse innerhalb des in der Fachinformation empfohlenen Überwachungszeitraums beobachtet wurden. Dabei wurden höhere Dosierungen (OR = 3,95) und männliches Geschlecht (OR = 0,42) als mögliche Risikofaktoren identifiziert, die eine Auftretenswahrscheinlichkeit des PDSS erhöhen könnten. Im Vergleich fanden sich in den Übersichtsarbeiten etwas niedrigere PDSS-Raten als zuvor in klinischen Studien beobachtet. Allerdings ist zu diskutieren, dass bisher nur Daten von Teilnehmenden ausgewertet wurden, die zuvor im Rahmen eines informierten Einwilligungsprozesses der Verarbeitung ihrer Gesundheitsdaten zugestimmt hatten, sodass die Möglichkeit eines Selektionsbias besteht. Demgegenüber liegen bisher keine sogenannte Real-World-Daten aus einer Therapieüberwachung der klinischen Regelversorgung vor.

## Real-World-Daten einer großen psychiatrischen Versorgungsklinik

Ziel der vorliegenden Arbeit war es daher, diese Forschungslücke durch Analyse einer Gesamtkohorte an einer großen regionalen Versorgungsklinik zu schließen und in den Kontext der bisherigen Fachliteratur einzuordnen. Methodisch wurde dafür eine Stichprobe anonymer Daten aller behandelten Personen erstellt, die in einem Indexzeitraum zwischen Januar 2020 (Beginn der Gabe von Olanzapinpamoat) bis einschließlich April 2024 an der Klinik für Psychiatrie, Psychotherapie und Psychosomatik der Universität Augsburg mit Olanzapinpamoat behandelt worden waren. Dabei konnten aufgrund der Anonymisierung der Daten keine Innersubjektvergleiche erfolgen, die Aussagen zum Risiko eines möglichen mehrfachen Auftretens eines PDSS erlauben würden. Stattdessen wurden alle Injektionen statistisch als unabhängige Ereignisse betrachtet. Ausgewertet wurden daher nur die Raten an PDSS innerhalb der Gesamtstichprobe ohne Aufschlüsselung etwaiger mehrfacher Ereignisse bei betroffenen Patientinnen und Patienten. Unterschieden wurden daneben unterschiedliche Dosierungen sowie das Behandlungssetting, im Sinne einer ambulanten Behandlung im Vergleich zum stationären Therapiesetting (Tab. 1).

**Tab. 1:** Verteilung der Dosierungen von Olanzapinpamoat aufgeschlüsselt nach Behandlungsjahren

*Dosisverteilungen ambulante Fälle*			
Jahr	210 mg	300 mg	405 mg
2020	0	0	4
2021	5	37	30
2022	1	49	43
2023	8	45	37
2024	4	39	55
*Dosisverteilungen stationäre Fälle*			
Jahr	210 mg	300 mg	405 mg
2020	4	13	18
2021	1	11	17
2022	0	28	8
2023	1	10	17
2024	3	2	6
*Prozentuale Verteilung (Gesamtstichprobe)*			
Jahr	210 mg	300 mg	405 mg
2020	10,3 %	33,3 %	56,4 %
2021	5,9 %	47,5 %	46,5 %
2022	0,8 %	59,7 %	39,5 %
2023	7,6 %	46,6 %	45,8 %
2024	6,4 %	37,6 %	56,0 %

Unsere Datenauswertung ergab, dass mit *n* = 139 Olanzapinpamoatinjektionen nur 28 % der Vergaben im stationären Setting erfolgten und die Mehrzahl (*n* = 357, 72 %) im ambulanten Setting durchgeführt wurde. Im ambulanten Setting wurden zudem mit *n* = 170 Injektionen (48 %) und *n* = 169 Injektionen (47 %) vor allem 300 mg bzw. 405 mg als Dosierungen verordnet. Dagegen wurden 210 mg Dosierungen in nur 5 % der Fälle (*n* = 18) verabreicht. Ganz ähnlich verhielt sich auch die Verteilung im stationären Setting. Hier machte die Dosierung von 210 mg nur 6 % (*n* = 9) der Injektionen aus, wohingegen 46 % (*n* = 64 Injektionen) und 47 % (*n* = 66 Injektionen) der Fälle 300 mg bzw. 405 mg als Dosierungen erhielten. Im gesamten oben genannten Erhebungszeitraum von 01/2020 bis 06/2024 trat bei insgesamt *n* = 496 Olanzapinpamoatinjektionen *n* = 1 PDSS-Event auf. Damit lag in unserer Stichprobe eine Inzidenzrate über die Gesamtkohorte von 0,2 % (1 Ereignis auf 496 Injektionen in der Gesamtstichprobe) vor. Wenngleich dies einer höheren Inzidenzrate als in der bisher vorliegenden Datenlage entspräche, fanden sich auf den Einzelfall betrachtet mit den bisherigen Daten vergleichbare kumulative Risikoraten, wie die nachfolgende Kasuistik belegt.

## Kasuistische Verlaufsbeschreibung

Im Fall der betroffenen Person handelte es sich um einen männlichen Patienten im Alter von 29 Jahren mit der Diagnose einer paranoiden Schizophrenie (ICD-10: F20.0), der im April 2024 eine Dosierung von 405 mg zum wiederholten Mal erhalten hatte. Die erste Gabe von Olanzapinpamoat war bei dem genannten Patienten bereits im Juni 2021 erfolgt. Seither waren die Termine zur Depotgabe durchgehend adhärent eingehalten worden, wobei je nach Ausmaß der Symptomlast an Positivsymptomen wechselnde Dosierungen zwischen 300 mg alle 2 Wochen und 405 mg alle 4 Wochen erfolgt waren. Bei der Verabreichung im April 2024 handelte es sich um die 57. Gabe in der genannten Behandlungsserie. Hieraus lässt sich über den Behandlungsverlauf des Patienten hinweg eine kumulative Risikorate von 1,75 % (1 Ereignis auf 57 Injektionen bei diesem Patienten) errechnen, was im Mittel vergleichbar ist mit den bisher veröffentlichten aus klinischen Studien und retrospektiven Übersichtsarbeiten. Die letzte Gabe davor war genau 28 Tage zuvor in gleicher Dosierung erfolgt. Wie alle Patientinnen und Patienten, die an unserem Zentrum mit Olanzapinpamoat behandelt werden, erhielt der Patient dabei die Injektion von einem für die Vergabe von Depotantipsychotika geschulten Behandlungsteam mittels tiefer glutealer Injektion, wobei ein Seitenwechsel des Verabreichungsorts im Vergleich zur vorherigen Gabe sichergestellt wurde. Im Nachgang der Vergabe wurde der Patient gemäß Fachinformation für einen Zeitraum von 3 h mit Hinblick auf seine Vitalparameter und Vigilanz überwacht. Etwa eine Stunde nach der Verabreichung des Depots um 10.00 Uhr morgens meldete sich der Patient spontan beim Pflegeteam und gab an, dass er sich schwach fühle und es ihm schlecht gehe. Klinisch fiel in der ärztlichen Untersuchung eine plötzlich eingetretene Hautblässe auf und der Patient erbrach sich. In den Vitalparametern zeigten sich ein leicht erhöhter Blutdruck mit 140/80 mm Hg, ein normaler Puls mit 75/min und eine normale O_2_-Sättigung mit 95 % unter Raumluft. Der Patient zeigte sich im Verlauf über 3 h hinweg immer wieder somnolent, jedoch durchgehend erweckbar. Er antwortete verlangsamt und leicht dysarthrisch, war jedoch im formalen Denken geordnet. Dazwischen traten immer wieder kurze psychomotorische Unruhezustände von wenigen Minuten Dauer auf, in denen der Patient versuchte aufzustehen oder sich aufzusetzen und dann wieder einschlief. Die Vitalwerte waren durchgehend stabil und eine Monitorüberwachung wurde nach Verlegung in die benachbarte internistische Klinik nicht als notwendig eingeordnet. In der Nacht nach dem Ereignis trat eine erhöhte Temperatur auf, die sich im Verlauf am Folgetag wieder komplett zurückbildete und kein laborchemisches Korrelat im Sinne von Entzündungsparametern zeigte. Die folgenden zwei Tage nach dem Ereignis bestanden anamnestisch noch eine erhöhte Müdigkeit und eine verwaschene Sprache, was am 3. Tag nach dem PDSS-Ereignis wieder vollständig abklang. Der Patient wünschte trotz des Ereignisses im weiteren Verlauf nach entsprechender Risiko- und Nutzenabwägung mit seinem Behandlungsteam die Fortführung der Olanzapindepotbehandlung und erhält seit Mai 2024 weiterhin eine Dosierung von 405 mg all 4 Wochen bei guter Verträglichkeit und klinischer Wirksamkeit. Wenngleich am Tag des PDSS-Ereignisses Blut abgenommen worden war, konnte im Nachgang keine Aussage über den Plasmaserumspiegel erfolgen, da eine Bestimmung des Medikamentenspiegels in der internistischen Klinik nicht erfolgt war.

## Zusammenfassung und Ausblick

Mit der vorliegenden Arbeit konnten erstmals Risikoraten basierend auf sogenannten Real-World-Daten im Sinne einer Vollerhebung in einem Indexzeitraum aus einer großen psychiatrischen Versorgungsklinik für das Risiko des Auftretens eines PDSS nach Injektion von Olanzapinpamoat der bisherigen Datenlage hinzugefügt werden. Dabei zeigten sich mit 1,75 % vergleichbare kumulative Risikoraten auf den Einzelfall bezogen wie diese auch mit 1,17 % bzw. 1,85 % in bisherigen Auswertungen berichtet worden waren. Aufgrund der geringen Anwendung der Formulierung in Deutschland zeigen die Daten auch bei tatsächlicher Anwendung im sektorübergreifenden Setting kein erhöhtes Risiko im Vergleich zu den bekannten Surveillance-Daten. Unsere Daten haben dabei den Vorteil aus der Vollerhebung, dass kein Verzerrungsfaktor im Sinne eines Nicht-Reportings eines PDSS vorliegt.

## Fazit für die Praxis

- Real-World-Daten zeigen vergleichbare kumulative Risikoraten des Olanzapin-Postinjektionssyndroms (PDSS).
- Beim Auftreten eines PDSS bilden sich Symptome insbesondere innerhalb der ersten Stunde aus.
- In den ersten 3 h sollte eine Überwachung der Vigilanz und Vitalparameter erfolgen.
- Da die Symptomatik häufig erst über einen Verlauf von 48–72 h wieder abklingt, sollte eine Überweisung in eine somatische Klinik zur Durchführung einer Überwachung erwogen werden.

## Interessenkonflikt

W. Strube hat Vortragshonorare von Mag & More (Neurocare), Recordati und Rovi erhalten. Er war Mitglied eines Advisory Boards von Recordati. E. Wagner war Mitglied von Advisory Boards von Recordati und Boehringer Ingelheim. A. Gartenmaier hat einen laufenden Dienstleistungsvertrag mit Janssen (Johnson & Johnson). A. Grünemeyer und K. Schmelzer haben jeweils keine Interessenskonflikte zu berichten. A. Hasan war Mitglied in Advisory Boards von Boehringer Ingelheim, Lundbeck, Janssen, Otsuka, Rovi und Recordati und hat Vortragshonorare von diesen Unternehmen sowie von AbbVie und Advanz erhalten. Er ist Herausgeber der deutschen S3-Leitlinie Schizophrenie. Einladungen zu Kongressen oder anderen Aktivitäten wurden von keinem der Autoren angenommen.
