# Erratum zu: Geringeres Risiko für Postinjektionssyndrome nach Vergabe von Olanzapin-Depot

**DOI:** 10.1007/s00115-024-01766-7

**Published:** 2024-11-07

**Authors:** Wolfgang Strube, Elias Wagner, Andreas Gartenmaier, Antje Grünemeyer, Klaus Peter Schmelzer, Alkomiet Hasan

**Affiliations:** 1 Klinik für Psychiatrie, Psychotherapie und Psychosomatik, Medizinische Fakultät, Universität Augsburg, 86156 Augsburg, Deutschland; 2 Evidenzbasierte Psychiatrie und Psychotherapie, Medizinische Fakultät, Universität Augsburg, Stenglinstraße 2, 86156 Augsburg, Deutschland; 3 Bezirkskliniken Schwaben, Apotheke des Bezirkskrankenhauses, Lindenallee 2, 89312 Günzburg, Deutschland; 4 Apotheke, Universitätsklinikum Augsburg, Stenglinstraße 2, 86156 Augsburg, Deutschland; 5 DZPG (Deutsches Zentrum für Psychische Gesundheit), Partnerstandort München/Augsburg, Augsburg, Deutschland

**Erratum zu:**

**Nervenarzt 2024**

10.1007/s00115-024-01733-2

Wir möchten die Leserinnen und Leser auf einen Fehler in unserem veröffentlichten Manuskript mit dem Titel „Geringeres Risiko für Post-Injektions-Syndrome nach Vergabe von Olanzapin-Depot“ [Ref.: Ms. No. DENE-D-24-00128] hinweisen, welches im Nervenarzt online veröffentlicht wurde. Wie beschrieben, wurde im letzten Satz des Abschnitts „Real World Daten einer großen psychiatrischen Versorgungsklinik“ fälschlich eine Inzidenzrate über die Gesamtkohorte von 0,002 % angegeben. Richtig ist jedoch eine Inzidenzrate von 0,20 % zu berichten. Wenngleich dies einer höheren Inzidenzrate als in der bisher vorliegenden Datenlage entspräche, fanden sich auf den Einzelfall unserer Kasuistik betrachtet niedrigere und mit den bisherigen Daten vergleichbare kumulative Risiko-Raten. Der von uns korrigierte Fehler führt daher zu einer Anpassung der Interpretation der Ergebnisse im Abschnitt „Zusammenfassung und Ausblick“. Hier lautete die ursprüngliche Aussage: „Dabei zeigten sich mit 0,002 % geringere Risikoraten, als mit 0,07 % bzw. 0,044 % in bisherigen Auswertungen berichtet worden waren“. Die korrigierte Aussage muss dagegen wie folgt formuliert sein: „Dabei zeigten sich mit 1,75 % vergleichbare kumulative Risikoraten für den Einzelfall (1 Ereignis bei 57 Injektionen), wie diese auch mit 1,17 % bzw. 1,85 % in bisherigen Auswertungen berichtet worden waren“. Analog sind auch der Abschnitt „Fazit für die Praxis“ sowie der Titel des Manuskripts wie folgt zu korrigieren: Der bisherige Titel „Geringeres Risiko für Post-Injektions-Syndrome nach Vergabe von Olanzapin-Depot“ ist zu ändern in „Risiko für Post-Injektions-Syndrome nach Vergabe von Olanzapin-Depot“. Im Fazit für die Praxis ist zudem die Herleitung „Real-World-Daten zeigen niedrigere Risikoraten des Olanzapin-Postinjektionssyndroms (engl. PDSS)“ zu korrigieren in „Real-World-Daten zeigen vergleichbare kumulative Risikoraten des Olanzapin-Postinjektionssyndroms (engl. PDSS)“. Die Korrektur verändert die Interpretation der Ergebnisse dahingehend, dass nun vergleichbare kumulative Risikoraten für das Auftreten eines Olanzapin-Postinjektionssyndroms (engl. PDSS) berichtet werden. Unsere vorherige Darstellung könnte demgegenüber dazu geführt haben, dass die Inzidenz des PDSS von Leserinnen und Lesern möglicherweise unterschätzt wurde. Unsere Richtigstellung könnte nun zu Verunsicherungen in der Leserschaft geführt haben. Wir möchten uns hierfür und für den nun entstehenden Mehraufwand entschuldigen und bedauern jegliche Unannehmlichkeiten, die dadurch entstanden sein könnten.

Der Originalbeitrag wurde korrigiert.

